# Characterization of engraftment dynamics in myelofibrosis after allogeneic hematopoietic cell transplantation including novel conditioning schemes

**DOI:** 10.3389/fonc.2023.1205387

**Published:** 2023-08-10

**Authors:** Sarah Jungius, Franziska C. Adam, Kerstin Grosheintz, Michael Medinger, Andreas Buser, Jakob R. Passweg, Jörg P. Halter, Sara C. Meyer

**Affiliations:** ^1^ Department of Biomedicine, University Hospital Basel and University of Basel, Basel, Switzerland; ^2^ Department of Biomedical Research, University of Bern, Bern, Switzerland; ^3^ Division of Hematology, University Hospital Basel, Basel, Switzerland; ^4^ Department of Hematology and Central Hematology Laboratory, Inselspital, Bern University Hospital, University of Bern, Bern, Switzerland

**Keywords:** myelofibrosis, myeloproliferative neoplasms, allogeneic hematopoietic cell transplantation, engraftment, reconstitution

## Abstract

**Introduction:**

Myelofibrosis (MF) is a rare hematopoietic stem cell disorder progressing to bone marrow (BM) failure or blast phase. Allogeneic hematopoietic cell transplantation (HCT) represents a potentially curative therapy for a limited subset of patients with advanced MF, who are eligible, but engraftment in MF vs. AML is delayed which promotes complications. As determinants of engraftment in MF are incompletely characterized, we studied engraftment dynamics at our center.

**Methods:**

A longitudinal cohort of 71 allogeneic HCT performed 2000–2019 with >50% after 2015 was evaluated.

**Results:**

Median time to neutrophil engraftment ≥0.5x109/l was +20 days post-transplant and associated with BM fibrosis, splenomegaly and infused CD34+ cell number. Engraftment dynamics were similar in primary vs. secondary MF and were independent of MF driver mutations in JAK2, CALR and MPL. Neutrophil engraftment occurred later upon haploidentical HCT with thiotepa-busulfan-fludarabine conditioning, post-transplant cyclophosphamide and G-CSF (TBF-PTCy/G-CSF) administered to 9.9% and 15.6% of patients in 2000-2019 and after 2015, respectively. Engraftment of platelets was similarly delayed, while reconstitution of reticulocytes was not affected.

**Conclusions:**

Since MF is a rare hematologic malignancy, this data from a large number of HCT for MF is essential to substantiate that later neutrophil and platelet engraftment in MF relates both to host and treatment-related factors. Observations from this longitudinal cohort support that novel conditioning schemes administered also to rare entities such as MF, require detailed evaluation in larger, multi-center cohorts to assess also indicators of long-term graft function and overall outcome in patients with this infrequent hematopoietic neoplasm undergoing allogeneic transplantation.

## Introduction

Allogeneic hematopoietic cell transplantation (HCT) is a potentially curative treatment for hematopoietic stem cell disorders including rare entities such as intermediate to high-risk myelofibrosis (MF). However, it is associated with significant morbidity and mortality, particularly early non-relapse mortality. Specifically, median time to hematopoietic engraftment appears to be later in MF (1–4) compared to acute myeloid leukemia (5–10), presumably due to pooling of infused stem cells by splenomegaly and/or an altered marrow microenvironment (11, 12). Prolonged cytopenia increases the patients’ risk for complications, particularly infections and bleeding events, as well as transfusion dependency, which has been associated with increased risk of relapse and higher mortality (13).

Tolerability of allogeneic HCT has substantially improved over recent years. Specifically, the advent of reduced-intensity conditioning (RIC) regimens has decreased peri-transplant complications and has made allogeneic HCT amenable for patients with higher age and/or comorbidities. This has increased the availability of allogeneic HCT for these populations, including the patients with intermediate to high risk MF, who are in important need of curative treatment options (14, 15). In addition, novel options of graft-vs-host-disease (GvHD) prophylaxis, such as e.g. with post-transplant cyclophosphamide (PTCy), have led to improved tolerability of haploidentical HCT, thus expanding the pool of potential HCT donors. While the number of haploidentical HCT in MF is still limited, emerging retrospective studies have assessed haploidentical HCT as overall safe (16–19). However, an influence on engraftment has not been thoroughly characterized in the setting of MF. Here, we sought to evaluate determinants of engraftment dynamics in MF upon allogeneic HCT in a longitudinal cohort of patients at our center, which also included first patients with preparatory regimens emerging more recently for MF.

## Methods

### Patient selection

We conducted a single-center retrospective study of engraftment dynamics in patients transplanted for primary or secondary MF. All patients undergoing allogeneic HCT at University Hospital Basel between 09/2000 and 09/2019 were screened. One single patient transplanted for MF with cord-blood as a stem cell source was excluded. Of 60 patients, 11 underwent a second HCT within the study period. Data was extracted from electronic patient files. The study was approved by the ethical committee Nordwest- und Zentralschweiz (EKNZ, no.2016-01930) and informed consent was obtained from all patients.

### Definitions

We evaluated determinants of time to engraftment for neutrophil, platelet and red cell lineages. Neutrophil engraftment was defined as first of 3 consecutive days with ≥0.5x10^9^/l neutrophils in peripheral blood, platelet engraftment as first of 3 consecutive days with ≥20x10^9^/l platelets in the absence of transfusions within the 7 preceding days, and red cell engraftment as first day with ≥30x10^9^/l reticulocytes. Conditioning was classified as reduced intensity (RIC) or myeloablative (MAC) conditioning according to Bacigalupo (20). Since thiotepa-busulfan-fludarabin (TBF) conditioning (thiotepa 10mg/kg, busulfan 6.4 mg/kg and fludarabine 150 mg/m^2^) was not included in this classification and consistently associated with haploidentical HCT at our center, it was evaluated separately. TBF was followed by haploidentical HCT, subsequent PTCy 50 mg/kg on days +3/+4 for GvHD prophylaxis and granulocyte colony-stimulating factor (G-CSF) support at 0.5 Mio units/kg from day+5 (TBF-PTCy/G-CSF) in this cohort. G-CSF support at 0.5 Mio units/kg/day was also applied to 5 patients with RIC and 1 patient with MAC regimens at variable time-points in the post-transplant course at the discretion of the treating physicians to promote reconstitution. Donor cell chimerism was assessed at 1, 3 and 6 months after HCT in peripheral blood and/or bone marrow. Poor graft function was evaluated as previously described (12, 21–24) with cytopenia in at least two hematopoietic lineages incl. neutrophils ≤ 1.5 G/l, platelets ≤ 30 G/l and/or Hb ≤ 85 g/l lasting for > 2 consecutive weeks following documented engraftment beyond d+14 in the presence of full donor chimerism and the absence of severe acute or chronic GvHD, relapse, drug-related or CMV reactivation-related myelosuppression. Patients’ disease risk category was determined according to respective prognostic scoring systems, primarily DIPSS plus (25), DIPSS (26), IPSS (27), and MYSEC (28) scores, as well as Cervantes (29) and MIPSS70 plus (30) scores in one patient each. Infections in the post-transplant period as well as thrombocyte concentrate (TC) and erythrocyte concentrate (EC) transfusion requirements were assessed in the first six months after allogeneic HCT.

### Statistical analysis

Descriptive statistics were used for patient and transplantation characteristics. Time to engraftment, time to full donor cell chimerism and overall survival were estimated by Kaplan-Meier method and log-rank test used to assess statistical significance between groups. Estimates are given as median with 95% confidence interval (CI). Patients were censored at death, secondary graft failure or second transplantation. Univariate Cox regression was used to evaluate spleen size as a continuous variable. Key disease characteristics such as splenomegaly and BM fibrosis and factors significant in univariate analysis were implemented in multivariate forward-conditional Cox regression. Results are given as hazard ratio (HR) with 95% CI. Cumulative incidence function was used for the estimation of non-relapse mortality (NRM) as well as for acute and chronic GvHD with relapse calculated as the competing event. P-values of ≤0.05 were considered statistically significant. Data were analyzed and figures generated with SPSS 28.0.0 (IBM, Armonk, NY, USA), GraphPad Prism 9.2.0 (GraphPad Inc., San Diego, CA, USA) and NCSS 2020 package (NCSS, LLC, Kaysville, Utah, USA).

## Results

### Patient and treatment characteristics

**Figure 1:**
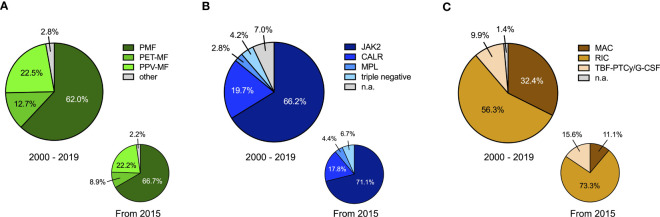
Characteristics of myelofibrosis patients undergoing allogeneic hematopoietic cell transplantation (HCT). **(A)** Subtype of myelofibrosis is indicated as primary myelofibrosis (PMF), post-essential thrombocythemia myelofibrosis (PET-MF), post-polycythemia vera myelofibrosis (PPV-MF) or other (including one patient with unclassifiable myeloproliferative neoplasm and one patient with MDS/MPN overlap syndrome) for the overall time-period 2000-2019 (main chart) and from 2015 onwards (small chart). **(B)** The frequencies of genetic driver mutations in JAK2, calreticulin (CALR) or the thrombopoietin receptor (MPL) as well as of triple negative cases non-mutated for JAK2, CALR and MPL are indicated for the overall time-period 2000-2019 (main chart) and from 2015 onwards (small chart). n.a. not available. **(C)** Proportions of the administered conditioning therapies for HCT in MF patients is indicated for the overall time-period 2000-2019 (main chart) and from 2015 onwards (small chart). MAC myeloablative conditioning, RIC reduced-intensity conditioning, TBF-PTCY thiotepa-busulfan-fludarabin conditioning followed by post-transplantation cyclophosphamide.

We retrospectively evaluated 71 allogeneic HCTs conducted in 60 MF patients in 2000–2019 with >50% performed after 2015 (**Table 1**, **Supplementary Table 1**). Median age at transplantation was 60 (31–72) years, increasing over time from 55.5 (31–61) to 58 (40–70), and 61 (34–72) years in the periods 2000–2009, 2010–2014, and 2015–2019, respectively, with a majority of patients being males. Median follow-up of survivors was 61 (24–198) months. Most patients (62.0%) suffered from primary MF (PMF), while 22.5% and 12.7% were transplanted for secondary MF evolving from polycythemia vera (PPV-MF) or essential thrombocythemia (PET-MF) and one patient each for unclassifiable myeloproliferative neoplasm (MPN-U) and MDS/MPN overlap syndrome (**Figure 1A**). *JAK2* V617F, calreticulin (*CALR*), and thrombopoietin receptor (*MPL*) driver mutations were detected in 66.2%, 19.7%, and 2.8% of patients, respectively, while 4.2% were triple negative for either mutation (**Figure 1B**). A majority of patients had intermediate-2 or high-risk disease (46.7% and 30%, respectively) as assessed by prognostic risk scoring, similarly in patients with PMF and secondary MF including PPV-/PET-MF (**Supplementary Figure 1**). BM fibrosis was advanced in most patients with 56.3% showing grade 3 fibrosis. Splenomegaly was prevalent with a median spleen size of 19 (11–32) cm at transplantation as assessed by sonography. Transplantations after previous splenectomy were uncommon with only 3 transplantations in 2 patients occurring in the setting of absent spleen. Ruxolitinib therapy before HCT was administered in a majority of 38/71 cases (53.5%) of the overall cohort with 30 patients receiving ruxolitinib up to transplantation and 8 patients with ruxolitinib discontinuation >1 month before HCT. Median time on ruxolitinib therapy before HCT was 10 months (range 1-78 months) and median spleen size at HCT did not significantly differ between ruxolitinib-treated and untreated patients (p=0.67). In our cohort, 7 patients received ruxolitinib after allogeneic HCT, mostly (5/7) after engraftment as a treatment of acute GvHD, while two patients with extensive splenomegaly were on ruxolitinib through the peri-transplant period. Myeloablative (MAC) or reduced-intensity (RIC) conditioning followed by cyclosporine and methotrexate or mycophenolate-mofetil with or without anti-thymocyte globuline (ATG) for GvHD prophylaxis was applied in 32.4% and 56.3% of allogeneic HCT, respectively. The novel preparatory regimen of haploidentical HCT with PTCy as GvHD prophylaxis, which beyond AML is also increasingly used in specific, rare entities such as in MF patients at our center, accounted for 15.6% of patients from 2015, but just 9.9% from 2000 (**Figure 1C**). In these patients, haploidentical HCT followed conditioning with thiotepa-busulfan-fludarabine, while subsequent PTCy on day +3/+4 was given for GvHD prophylaxis and granulocyte colony-stimulating factor (G-CSF) from day+5 for regeneration support (TBF-PTCy/G-CSF). Median number of infused CD34+ cells was 7.2 (1.94–18.8) x10^6^/kg body weight. Peripheral blood stem cells (PBSC) represented the prevalent CD34+ cell source (87.3%), while a minority of non-haploidentical (6.3%) and a majority of haploidentical HCTs (57%) used BM as source. Second allogeneic HCT was performed in 11 patients to manage graft failure, relapse or persistent disease.

**Table 1:** Baseline characteristics of patients’ allogeneic hematopoietic cell transplantations for myelofibrosis.

Patient and transplantation characteristics	Number of transplantations n (%) unless otherwise specified	
Time period		
2000-2009	6 (8.5)	
2010-2014	20 (28.2)	
2015-2019	45 (63.4)	
Sex		
male	43 (60.6)	
female	28 (39.4)	
Age at transplantation		
<55 years	20 (28.2)	
55 - 64 years	32 (45.1)	
>64 years	19 (26.8)	
*median age (range)*	60.0 (31 - 72) years	
Diagnosis		
PMF	44 (62.0)	
Secondary MF	25 (35.2)	
* PET-MF*	*9 (12.7)*	
*PPV-MF*	*16 (22.5)*	
MPN/MDS overlap syndrome	1 (1.4)	
MPN-U	1 (1.4)	
Driver mutation		
JAK2 V617F	47 (66.2)	
CALR	14 (19.7)	
MPL	2 (2.8)	
triple negative	3 (4.2)	
n.a.	5 (7.0)	
Fibrosis grade		
grade 1	2 (2.8)	
grade 2	15 (21.1)	
grade 3	40 (56.3)	
n.a.	14 (19.7)	
Spleen		
splenomegaly (≥13 cm)	65 (91.5)	
no splenomegaly	3 (4.2)	
prior splenectomy	3 (4.2)	
*median size (range)*	19 (11 - 32) cm	
Ruxolitinib before HCT		
no	33 (46.5)	
yes	38 (53.5)	
*median time (range)*	10 (1 - 78) months	
Donor relation		
matched related	26 (36.6)	
haploidentical	7 (9.9)	
matched unrelated	35 (49.3)	
mismatched unrelated	2 (2.8)	
n.a.	1 (1.4)	
Stem cell source		
PBSC	62 (87.3)	
BM	8 (11.3)	
n.a.	1 (1.4)	
Stem cell dose		
<6 x10^6^/kg	30 (42.3)	
6 - 8 x10^6^/kg	15 (21.1)	
>8 x10^6^/kg	24 (33.8)	
n.a.	2 (2.8)	
*CD34+ cells (range)*	7.2 (1.94 - 18.8) x10^6^/kg	
Conditioning regimen		
RIC (FluBu, FluTBI, FluMel)	40 (56.3)	
MAC (CyBu, CyTBI)	23 (32.4)	
TBF	7 (9.9)	
n.a.	1 (1.4)	
GvHD prophylaxis		
CyA MTX +/- ATG	59 (83.1)	
CyA MMF +/- PTCy	10 (14.1)	
CyA	1 (1.4)	
n.a.	1 (1.4)	
ATG		
no	25 (35.2)	
yes	45 (63.4)	
n.a.	1 (1.4)	
PTCy		
no	63 (88.7)	
yes	7 (9.9)	
n.a.	1 (1.4)	
G-CSF		
no	57 (80.3)	
yes	13 (18.3)	
n.a.	1 (1.4)	
CMV risk		
D-/R-	27 (38.0)	
D+/R+	20 (28.2)	
D-/R+	14 (19.7)	
D+/R-	9 (12.7)	
n.a.	1 (1.4)	
Blood-group barrier		
no	48 (67.6)	
minor	4 (5.6)	
major	13 (18.3)	
bidirectional	5 (7.0)	
n.a.	1 (1.4)	

Characteristics of patients undergoing a total of 71 allogeneic hematopoietic cell transplantations in 2000-2019 are indicated in absolute numbers with relative proportions in brackets. Continuous variables are indicated as median and range in brackets. n.a., not available; PMF, primary myelofibrosis; MF, myelofibrosis; PPV-/PET-MF, post-polycythemia vera/post-essential thrombo-cythemia myelofibrosis; MPN-U, unclassifiable myeloproliferative neoplasm; PBSC, peripheral blood stem cells; BM, bone marrow; RIC, reduced-intensity conditioning; MAC, myeloablative conditioning; TBF, thiotepa-busulfan-fludarabin conditioning; GvHD, graft versus host disease; CyA, cyclosporine A; MTX, methotrexate; MMF, mycophenolate mofetil; ATG, anti-thymocyte globulin; PTCy, post-transplantation cyclophosphamide; G-CSF, granulocyte colony-stimulating factor; CMV, cytomegalovirus; D/R, donor/recipient

### Engraftment in first vs. second transplantations

**Figure 2:**
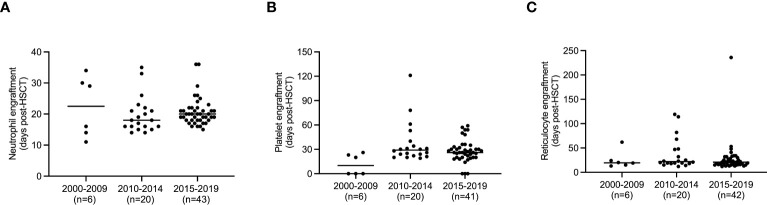
Time to engraftment in hematopoietic lineages in different time periods. **(A)** Time to engraftment of neutrophils after hematopoietic cell transplantation (HCT) for myelofibrosis (MF) defined as the first of 3 consecutive days with ≥0.5x10^9^/l neutrophils in peripheral blood is indicated for early (2000-2009), intermediate (2010-2014) and recent (2015-2019) time periods. **(B)** Time to engraftment of platelets after HCT for MF defined as the first of 3 consecutive days with ≥20x10^9^/l platelets in the absence of transfusions within the 7 preceding days is indicated for early (2000-2009), intermediate (2010-2014) and recent (2015-2019) time periods. **(C)** Time to red cell engraftment after HCT for MF defined as the first day with ≥30x10^9^/l reticulocytes is indicated for early (2000-2009), intermediate (2010-2014) and recent (2015-2019) time periods. The number of performed HCT in each time period is shown in brackets, patients who failed to engraft the respective lineage are not displayed.

**Figure 3:**
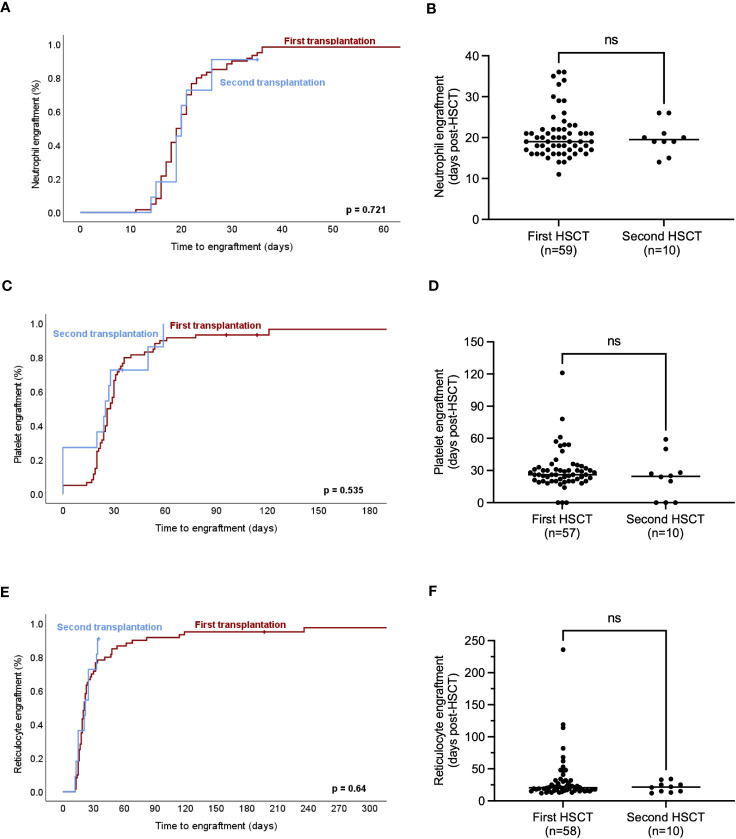
Engraftment dynamics in hematopoietic lineages after first and second hematopoietic cell transplantation (HCT). **(A)** Proportion of neutrophil engraftment over time is shown for first as compared to second HCT for MF. **(B)** Time to engraftment of neutrophils in peripheral blood is similar in first and second HCT in MF. **(C)** Proportion of platelet engraftment over time is shown for first as compared to second HCT for MF. **(D)** Time to engraftment of platelets in peripheral blood is similar in first and second HCT in MF. **(E)** Proportion of red cell engraftment over time is shown for first as compared to second HCT for MF. **(F)** Time to red cell engraftment of platelets in peripheral blood is similar in first and second HCT in MF. Median and individual data points are indicated and compared by student’s t-test with p-values ≤0.05 considered statistically significant **(B, D, F)**. Kaplan-Meier estimates were assessed by log-rank test **(A, C, E)**. ns, non-significant.

Given that hematopoietic engraftment, which is known to be delayed in MF vs. AML (4, 10), impacts on the susceptibility for infectious and bleeding complications, we evaluated factors influencing engraftment in the neutrophil, megakaryocytic and erythroid lineages. Neutrophil engraftment occurred at a median of 20 (11–36) days post-transplant, while one patient experienced graft failure and one deceased before engraftment. Time to neutrophil engraftment was similar over the different time periods 2000-2009, 2010-2014 and 2015-2019 (p=0.374, **Figure 2A**) and was analogous in first and second HCTs (p=0.72, **Figures 3A, B**). Median time to platelet engraftment was 26 (0–121) days, including 6 patients maintaining platelets ≥20x10^9^/l throughout the peri-transplantation period. Platelet engraftment was faster in 2000–2009 vs. later periods (p<0.001), but remained stable 2010–2014 and 2015–2019 (p=0.931, **Figure 2B**), and was analogous in first and second transplantations (p=0.535, **Figures 3C, D**). Reticulocyte engraftment occurred at a median of 21 (12–236) days with no relevant difference among the three time periods (p=0.687, **Figure 2C**) or between first and second transplantations (p=0.64, **Figures 3E, F**). Donor cell chimerism 1, 3 and 6 months after HCT was similar in 2010-2014 and 2015-2019, while lower 2000-2009 at 1-3 months, and was analogous in first and second HCTs (**Supplementary Figures 2**, **3**). Given that hematopoietic reconstitution times in all three lineages were analogous between first and second transplantations, as well as the limited number of second transplantations, first and second transplants were pooled for further analyses.

### Engraftment relates to MF- and transplant-related factors

**Figure 4:**
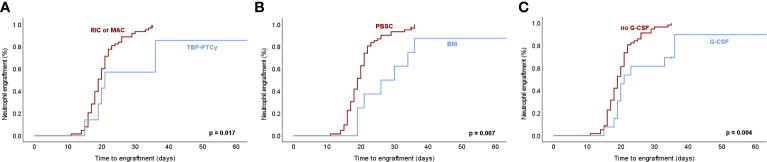
Neutrophil engraftment dynamics in association with transplant-related factors in myelofibrosis. **(A)** Neutrophil engraftment after hematopoietic cell transplantation (HCT) for myelofibrosis (MF) defined as the first of 3 consecutive days with ≥0.5x10^9^/l neutrophils in peripheral blood associates significantly with thiotepa-busulfan-fludarabine conditioning followed by post-transplant cyclophosphamide (TBF-PTCy) as a preparatory regimen of HCT as compared to reduced-intensity (RIC) or myeloablative (MAC) conditioning therapies. **(B)** Bone marrow (BM) as CD34+ cell source used in a majority of TBF-PTCy haploidentical HCT associates with later neutrophil engraftment. **(C)** Post-transplant G-CSF, which is administered in all TBF-PTCy haploidentical HCT as well as in a minority of HLA-matched HCT associated with later neutrophil engraftment. Kaplan-Meier estimates were assessed by log-rank test with p-values ≤0.05 considered statistically significant. PBSC, peripheral blood stem cells; G-CSF, granulocyte-colony stimulating factor.

To explore potential determinants of engraftment, we evaluated MF disease characteristics including primary vs secondary MF, driver mutations in JAK2, CALR and MPL, prognostic risk group, splenomegaly and BM fibrosis as well as ruxolitinib therapy before HCT. Transplant-related factors with potential impact on engraftment dynamics were also assessed including the type of conditioning regimen, GvHD prophylaxis, type of donor, CD34+ cell number and source, administration of G-CSF support, and donor-recipient relation for blood group and CMV status. PMF vs secondary MF (median 20 vs 19 days; p=0.65), driver mutation status (p=0.57) as well as prognostic risk group (p=0.21) did not influence kinetics of neutrophil regeneration. In line with the notion that alterations of the microenvironment could interfere with engraftment (11), we observed that BM fibrosis significantly influenced neutrophil engraftment (median 15 vs 20 days, grade 1 vs 3, p=0.017; median 15 vs 19 days, grade 1 vs 2, p=0.054; **Supplementary Table 2**). In addition, splenomegaly impacted on engraftment of the neutrophil lineage as reflected by reconstitution times significantly relating to spleen size by univariate Cox-regression analysis (p=0.032, **Supplementary Table 2**). The shortened engraftment times observed in the setting of splenectomy should be interpreted with caution given that only a small minority of 3 transplantations in 2 MF patients of the entire cohort were performed after a splenectomy based on the rarity of this intervention. Ruxolitinib therapy before HCT did not significantly impact on neutrophil engraftment in our cohort (p=0.27). CD34+ cell dose significantly influenced neutrophil engraftment with a median of 18 days if >8x10^6^ cells/kg were infused versus 20 days with low cell numbers <6x10^6^ cells/kg (p=0.037) consistent with effects observed in similar cohorts (31, 32). We explored a potential impact of conditioning schemes used as preparatory regimens for allogeneic HCT in MF. We did not observe a difference in time to neutrophil engraftment for RIC vs MAC protocols (p=0.819). However, a potential effect of TBF conditioning, which was used exclusively for haploidentical HCT in our cohort, was detectable with TBF associated with later neutrophil engraftment (median 21 vs 19 days, p=0.017; **Figure 4A**). This finding should be cautiously noted given the low absolute number of TBF-conditioned haploidentical HCT in this longitudinal cohort, and should promote evaluations in larger, multicentric cohorts of MF undergoing HCT. While ATG as GvHD prophylaxis related to shortened engraftment (p=0.028), PTCy which was exclusively used for TBF-conditioned haploidentical HCT in our cohort, associated with longer neutrophil reconstitution times. This was similarly reflected in HCT from haploidentical as compared to HLA matched donors (21 vs 20 days, p=0.019), while matched related and unrelated donors behaved analogously (p=0.176). BM as CD34+ cell source, which was used in the majority of haploidentical TBF-PTCy HCTs, and post-transplant G-CSF consistently used in TBF-PTCy HCTs in our cohort, analogously associated with later engraftment of neutrophils (median 26 vs 19 days, p=0.007 for BM vs. PBSC; median 21 vs 19 days, p=0.004 for post-transplant G-CSF; **Figures 4B, C**). Given that TBF conditioned haploidentical transplants overlap with the use of BM as a stem cell source and administration of G-CSF support, these findings may reflect an overall different behavior of engraftment dynamics with this procedure.

**Figure 5:**
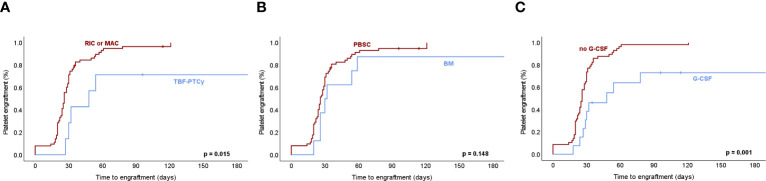
Platelet engraftment dynamics in association with transplant-related factors in myelofibrosis. **(A)** Platelet engraftment after hematopoietic cell transplantation (HCT) for myelofibrosis (MF) defined as the first of 3 consecutive days with ≥20x10^9^/l platelets in the absence of transfusions within the 7 preceding days associates significantly with thiotepa-busulfan-fludarabine conditioning followed by post-transplant cyclophosphamide (TBF-PTCy) as a preparatory regimen of HCT as compared to reduced-intensity (RIC) or myeloablative (MAC) conditioning therapies. **(B)** Bone marrow (BM) as CD34+ cell source used in a majority of TBF-PTCy haploidentical HCT associates with later platelet engraftment. **(C)** Post-transplant G-CSF, which is administered in all TBF-PTCy haploidentical HCT as well as in a minority of HLA-matched HCT associated with later platelet engraftment. Kaplan-Meier estimates were assessed by log-rank test with p-values ≤0.05 considered statistically significant. PBSC, peripheral blood stem cells; G-CSF, granulocyte-colony stimulating factor.

Similar to the findings for neutrophil engraftment, reconstitution of platelets did not reveal differential effects in primary vs secondary MF, with JAK2, CALR and MPL driver mutations or different prognostic risk group, but splenomegaly significantly prolonged platelet engraftment (p=0.002, HR: 0.930, **Supplementary Table 2**). Consistent with an impact of splenomegaly, the few HCT after previous splenectomy associated with earlier platelet engraftment (p<0.001). Similar to the influence of BM fibrosis on neutrophil regeneration, fibrosis grade also showed a trend for later platelet engraftment (p=0.088). Ruxolitinib therapy before HCT did not show a significant effect (p=0.07). As for transplant-related factors, RIC and MAC regimens led to comparable engraftment dynamics (p=0.579), while later platelet engraftment was observed upon TBF conditioning with PTCy as GvHD prophylaxis (p=0.015; **Figure 5A**) and administration of post-transplant G-CSF (p=0.001), while BM vs PBSC showed a similar trend (p=0.148; **Figures 5B, C**). Higher CD34+ cell doses significantly correlated with faster engraftment (p=0.021, **Supplementary Table 2**) as described (11), while blood-group barrier and CMV serology status did not influence platelet reconstitution. Overall, red cell engraftment as reflected by reticulocytes ≥30x10^9^/l was less dependent on the evaluated determinants as compared to neutrophil and platelet lineages including prognostic risk groups and pre-transplant ruxolitinib treatment (p=0.97). However, red cell engraftment still related to splenomegaly, which prolonged engraftment time (p=0.008, HR: 0.942; **Supplementary Table 2**). In line with the notion that not only regeneration but also consumption by e.g. hemolysis might affect reconstitution of the red cell mass, we observed later reticulocyte engraftment upon major (p=0.032), minor (p=0.013) and bidirectional (p=0.047) blood group barriers.

**Figure 6:**
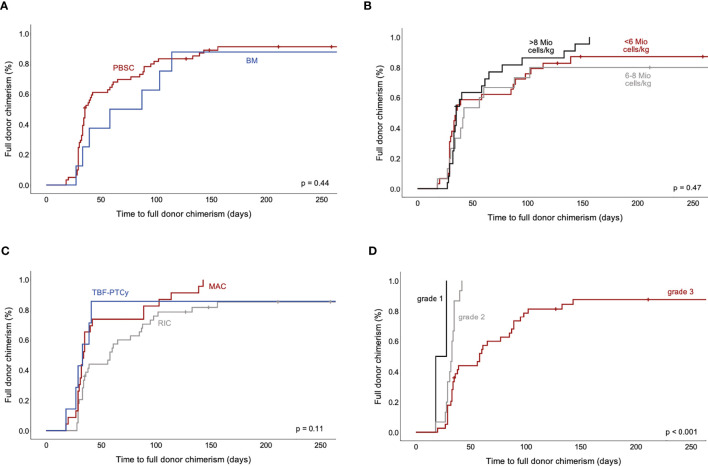
Time to full donor cell chimerism after allogeneic HCT for MF. Impact of selected factors on development of full donor cell chimerism are displayed. Time to 100% donor cell chimerism in peripheral blood did not show significant differences for CD34+ cell source **(A)** and cell number **(B)** nor for type of conditioning regimen **(C)**. More extensive bone marrow fibrosis associated with later establishment of full donor cell chimerism **(D)**. HCT, hematopoietic cell transplantation; MF, myelofibrosis.

Development of full donor cell chimerism established at a median of 35 days after HCT (range 18-156 days), did not show significant differences relating to CD34+ cell source and number, type of conditioning regimen, use of ATG or PTCY as well as G-CSF, donor/recipient constellation or pre-transplant ruxolitinib therapy (**Figure 6**). Similarly, disease-specific factors including MF subtypes PMF and PET-/PPV-MF, driver mutation status or splenomegaly did not significantly impact on the dynamics of full chimerism development, whereas higher fibrosis grade associated with increased time to full chimerism (median 58 days, 33 days and 18 days for grade 3, grade 2 and grade 1 fibrosis, respectively, p<0.001, **Figure 6**).

### Multivariate analyses of novel conditioning schemes

To consolidate these findings, we performed multivariate analyses, including age at transplantation, gender, MF disease characteristics, and factors with significant effects in univariate analysis. Upon correction for splenectomy, fibrosis grade, conditioning, use of ATG, G-CSF, donor type, CD34+ cell dose and source, age and gender, an impact of splenectomy on neutrophil engraftment was confirmed, although only 3 transplantations in 2 patients were performed after previous splenectomy in our cohort (p<0.001, HR: 107.67). Given the rarity of splenectomy in our cohort (4.2%), conflicting data on its effects on outcome (33, 34), and decreasing relevance of this procedure, we omitted splenectomy as a factor from further multivariate analyses. Consequently, post-transplant G-CSF support (p=0.02, HR: 0.415) and BM as CD34+ cell source (p=0.039, HR: 0.421) were confirmed to associate with later neutrophil engraftment (**Supplementary Tables 3**, **4**). However, this finding should be cautiously interpreted considering the substantial overlap of these factors with TBF-conditioned haploidentical HCT in our cohort and the limited number of patients, although substantial for a single center longitudinal cohort of higher risk, transplant-eligible MF.

Similarly, multivariate analysis for determinants of platelet engraftment confirmed the effects of post-transplant G-CSF administration (p<0.001, HR: 0.273), grade 3 vs grade 1 BM fibrosis (p=0.013, HR: 0.145) and splenectomy (p=0.011, HR: 7.044) as seen in univariate analysis. When splenectomy, which was only performed in 2 patients, was omitted from the model, post-transplant G-CSF (p=0.002, HR: 0.301) and grade 3 vs grade 1 BM fibrosis (p=0.026, HR: 0.179) maintained significant effects along with splenomegaly (p=0.021, HR: 0.94, **Supplementary Tables 3**, **4**) in line with similar cohorts (11, 32). For erythroid reconstitution, multivariate analyses confirmed the influence of a major blood group barrier (p=0.019, HR: 0.449) and splenomegaly (p=0.012, HR: 0.942) on reticulocyte engraftment dynamics. Factors associated with haploidentical TBF-conditioned HCT were not evident, confirming the finding from univariate analyses that red cell reconstitution is not as much impacted by novel preparatory regimens (**Supplementary Tables 3**, **4**).

### Longer-term outcomes including poor graft function

To explore potential long-term effects of delayed engraftment, we assessed overall survival (OS) in MF after first allogeneic HCT, which was 6.7 years (2460 days) in our cohort in line with previous studies (35–38). Patients with neutrophil engraftment later than the median showed a trend towards reduced OS without statistical significance (median OS 2.7 years, 95% CI 0-5.8 years, p=0.099), while platelet or red cell engraftment after the median did not impact on OS in our cohort. A potential impact of delayed engraftment on OS after second allogeneic HCT was not assessed given the limited number of patients. The cumulative incidence of non-relapse mortality (NRM) in our cohort at 5 years after first HCT was 19.3% and not significantly different in patients with delayed engraftment when assessed as engraftment time greater than the median of the cohort (95% CI 11.3-33.2%, p=0.84, **Supplementary Figure 4**). Cumulative incidence of relapse in the overall cohort was 26.9% at 5 years (95% CI 17.7-40.9%). Acute graft versus host disease (GvHD), as assessed for grades 2-4, occurred at a cumulative incidence of 33.1% at 180 days after HCT without a significant difference upon delayed engraftment (95% CI 23.7-46.2%, p=0.48, **Supplementary Figure 5**), while chronic GvHD was documented in 46.7% (95% CI: 35.7-61.3%) at 5 years after HCT and was similar in patients with delayed neutrophil engraftment (p=0.28, **Supplementary Figure 6**). Infections within 6 months post-transplant were assessed from 2010 onwards, as only a limited number of transplantations (n=6) were performed 2000-2009. While a majority of patients suffered from post-transplant infections in 2010-2014 and 2015-2019 (80.0% and 64.4%, respectively), patients with delayed neutrophil engraftment when assessed as engraftment time greater than the median of the cohort, showed similar rates of post-transplant infections as compared to patients with engraftment time shorter than the median in both time periods (75.0 vs. 83.3% and 56.5% vs. 70.0%, respectively). Within 6 months after HCT, a median of one infection per patient occurred with a range of 0-5 post-transplant infections per patient similarly in patients with delayed engraftment (**Supplementary Figure 7**).

Notably, delayed engraftment impacted on transfusion requirements after allogeneic HCT. We observed that thrombocyte concentrate (TC) transfusion dependence at day 100 after HCT was > 5-fold more prevalent upon delayed platelet engraftment when assessed as engraftment time longer than the median of the cohort (20% vs. 3% of patients). While delayed neutrophil engraftment also related to somewhat higher TC transfusion dependence at day 100 (15.2% vs. 9.7%), delayed reticulocyte engraftment did not have an impact. For erythrocyte concentrate (EC) transfusion dependence at day 100 after HCT, we observed that it was not affected by delayed neutrophil engraftment (35.5% vs. 36.4%), but increased upon prolonged reticulocyte and platelet engraftment (50.0% vs. 21.2% EC transfusion dependent upon delayed red cell engraftment and 42.9% vs. 24.2% upon delayed platelet engraftment, respectively). Given these findings, we specifically assessed the total number of TC and EC transfusions until engraftment and found that the number of required TC transfusions was significantly higher upon delayed platelet engraftment (median 29 vs. 6 TC, p < 0.0001, **Supplementary Figures 8A-C**). The number of required EC transfusions was significantly increased in settings of delayed reticulocyte (median 18 vs. 8 EC, p < 0.0001) and also delayed platelet engraftment (median 16.5 vs. 8 EC, p = 0.0006, **Supplementary Figures 8D-F**). We confirmed these findings over a more extended time period of 6 months after HCT suggesting a relevance of engraftment dynamics for transfusion requirements for such a longer period. We found that the number of TC transfusions within 6 months after HCT was significantly increased in settings of delayed platelet (median 32 vs. 7 TC, p <0.0001) and also neutrophil engraftment (median 23.5 vs. 15 TC, p = 0.04, **Supplementary Figures 9A-C**). Four patients did not engraft platelets and required 86-200 TC transfusions within 6 months after HCT (not shown). For EC, the number of required transfusions within 6 months after HCT was higher upon delayed reticulocyte (median 24 vs 11 EC, p < 0.0001) as well as platelet engraftment (median 23 vs. 10.5 EC, p < 0.0001), but not affected by neutrophil engraftment dynamics (**Supplementary Figures 9D-F**). Three patients did not engraft reticulocytes and required 24-58 EC transfusions within 6 months after HCT (not shown).

In addition, poor graft function as previously defined (12, 21–24) was observed in 8 patients within 6 months after HCT. Median onset was at 39 days (range 29-95 days) after HCT and lasted for a median duration of 58 days (range 21-203 days). In all but one patient all three hematopoietic lineages incl. neutrophils, platelets and reticulocytes were affected (**Supplementary Table 5**). Median age of patients with poor graft function was similar to the entire cohort (64 years, range 46-70 years). A majority of 7/8 patients were transplanted for PMF (88%) and calreticulin mutations were overrepresented as driver mutations in 5/8 (63%) patients. Splenomegaly was observed in all patients with poor graft function at median spleen size of 18 cm (range 15-27 cm) at HCT similar to the overall cohort. Bone marrow fibrosis was pronounced with grade 3 fibrosis in most patients. Infused CD34+ cells were predominantly of peripheral origin and median dose was somewhat lower as compared to the entire cohort at 6.1 Mio/kg body weight. A major donor-recipient blood group barrier was present in 5/8 patients with poor graft function and thus more prevalent than in the overall cohort (63%).

## Discussion

Allogeneic HCT represents the sole treatment option with curative potential for patients with higher risk MF, a rare hematologic neoplasm with patients in high need for effective therapeutic approaches (39). The subset of intermediate and high risk MF patients eligible for allogeneic HCT represents a limited population, which might be challenging to study at larger numbers. However, studies on transplantation approaches specifically in MF are essentially required since MF patients display characteristics, which are different from other myeloid malignancies and pose particular challenges to successful allogeneic HCT. Particularly, hematopoietic cell engraftment in MF has been shown to be compromised by adverse BM microenvironment features such as e.g. BM fibrosis, as well as through cell pooling by the prevalent splenomegaly (11, 12, 32), thus mediating an increased risk for post-transplant infections, bleeding and iron overload in MF. Here we report on the factors relating to engraftment dynamics in a longitudinal patient cohort of MF patients after allogeneic HCT between 2000 and 2019 at a major transplant center in Switzerland to provide additional evidence on the aspect of delayed engraftment in the neutrophil, platelet and red cell lineage in this infrequent patient group. While the overall cohort has a substantial size of 60 patients undergoing a total of 71 transplantations, the subsets of patients evaluable for specific factors relating to recent developments such as e.g. novel conditioning schemes may still be limited in number given the rarity of MF patients with high risk features and eligibility for HCT. However, we believe it is instrumental that these findings are reported timely to provide a basis and promote further evaluations in larger, multi-centric studies of MF.

Since engraftment dynamics in MF after allogeneic HCT are incompletely characterized, we evaluated potential determinants of reconstitution in the neutrophil, platelet and erythroid lineages. Median time to neutrophil engraftment in our MF cohort was comparable to other studies of allogeneic HCT in MF such as e.g. Kunte et al. reporting engraftment at a median of 20 days after haploidentical HCT (18). However, since PBSC were used as cell source in the vast majority of transplants (>85%) in that study, comparability to our cohort of transplanted MF patients is limited. Regarding platelet engraftment, variable definitions are used hampering comparisons across studies, and red cell engraftment as reflected by reticulocytes has only been reported by one study using a different cutoff (40). While engraftment failures were rare in our cohort, we assessed determinants of engraftment dynamics, which associated with later reconstitution in neutrophil, platelet, and erythroid lineages. The significance of key factors known to impact on engraftment in MF, such as splenomegaly or BM fibrosis, were evident in our study concordant with similar cohorts, thus highlighting the validity of our cohort as compared to other reports (11, 32). First, splenomegaly associated with later engraftment of neutrophils, platelets, and reticulocytes, highlighting that the prevalent finding of enlarged spleen warrants attention upon transplantation for MF. In addition, we observed significantly faster engraftment of neutrophil, platelet, and red cell lineages after pre-transplant splenectomy, although only 3 HCT in 2 splenectomized patients were performed. Similar findings were also reported by Polverelli et al., which included faster engraftment after splenectomy and delayed engraftment upon gross splenomegaly (33). At the time of HCT, patients with and without ruxolitinib therapy showed similar spleen size and ruxolitinib therapy, which was given in a majority before HCT, did not significantly influence engraftment dynamics. In line with the notion that BM fibrosis contributes to a microenvironment, which hinders engraftment (11), we observed longer reconstitution times for neutrophils and platelets in settings with higher-grade BM fibrosis as well as later establishment of full donor cell chimerism. It should be noted though that since MF is transplanted in advanced phases of the disease, sample size for grade 1 fibrosis was small. Of note, the disease-modifying potential of therapies including the reduction of BM fibrosis, represents an increasing interest of clinical studies for novel therapeutic approaches with targeted inhibitors in MF. Thus, such novel agents or combination therapies might represent suitable options as bridging therapy to allogeneic HCT, which would favor engraftment through pre-transplant reduction of BM fibrosis.

As to transplant-related factors, a correlation between CD34+ cell dose and neutrophil engraftment has been reported and was also evident in our cohort (31, 32). For red cell reconstitution, it has been established that engraftment may be delayed upon major blood group barriers but not in settings of minor, bidirectional or without ABO mismatch (41, 42). This has been attributed to residual circulating antibodies mediating hemolysis of donor-derived red cells, which was also found to prolong erythrocyte but not neutrophil engraftment in this study (43). In regard to preparatory regimens, reduced intensity conditioning (RIC) was most prevalent in the entire cohort and was applied to the vast majority of patients transplanted after 2015. Refinement of conditioning therapy such as by RIC approaches has largely improved tolerability of allogeneic HCT also in MF similarly to other hematologic malignancies and such modified conditioning regimens are widely used (44–47). More recently, the advent of TBF conditioning followed by PTCy as GvHD prophylaxis has improved the tolerability of haploidentical HCT and has enlarged the donor pool. Thus, TBF-conditioned haploidentical HCT with PTCy was also increasingly used in MF patients at our center. However, engraftment upon haploidentical HCT is incompletely characterized for MF so far, which relates at least in part to the challenges of studying new approaches in populations as rare as advanced, transplant-eligible MF. So far, two studies focused on haploidentical/mismatched family donors and found engraftment rates >90% (17, 18). Bregante et al. analyzed matched related, unrelated and alternative donors including mismatched family donors, and found improved engraftment rates after introduction of TBF conditioning (16). Battipaglia et al. reported lower engraftment rates at prolonged times with haploidentical donors, but without implications for survival outcomes (19), while Angelucci et al. related reduced survival to graft failure in haploidentical transplants for MF (48). TBF conditioning followed by PTCy as GvHD prophylaxis has been specifically used as a preparatory regimen for haploidentical HCT. We observed that HCT after TBF-PTCy as a preparatory regimen showed slower reconstitution dynamics for neutrophils and platelets in our MF cohort and this same finding also related to the donor type with delayed regeneration in HCT from haploidentical donors, which converge with the TBF-PTCy regimen at our center. Similarly, later engraftment associating with BM as a CD34+ cell source or use of G-CSF support may be in part driven by these effects, since haploidentical TBF-PTCy HCT was overrepresented both among BM transplants and transplants with G-CSF support. These observations are supported by Battipaglia et al., who showed delayed neutrophil engraftment with haploidentical stem cell grafts (19), and Ballen et al. who described lower engraftment rates in patients receiving grafts from partially matched or mismatched family donors (49). In contrast, we found no differential effects on engraftment when RIC or MAC preparatory regimens were used. While our study highlights that haploidentical TBF-PTCy HCT is feasible in MF, a differential impact on engraftment dynamics in both the neutrophil and platelet lineages might be at play and should be evaluated in the appropriate larger-scale, multi-center studies to allow for conclusive assessments of such effects specifically in HCT for MF given the risks relating to later reconstitution and prolonged neutropenia and thrombocytopenia.

Inherent limitations of our study primarily relate to the number of MF patients undergoing allogeneic HCT. Although overall it is substantial for a single transplant center given the rarity of higher risk MF eligible for HCT, it remains rather limited when specific aspects such as e.g. influences of more recent conditioning schemes should be assessed. In addition, multivariate analyses of previously reported determinants of post-transplant engraftment such as e.g. splenomegaly and BM fibrosis may be compromised by the limited patient numbers. However, since post-transplant reconstitution in MF is prolonged as compared to other myeloid malignancies, it is imperative to initiate studies of factors, which could further compromise reconstitution and to highlight the need for larger studies which will require collaborative efforts in the field. A second limitation concerns the convergence of the TBF-PTCy treatment with haploidentical donor HCT at our center, which also partly overlaps with the use of BM as stem cell source and G-CSF support. The strong association of TBF-conditioned haploidentical HCT and PTCy in our cohort hinders a dissection of which factor(s) would interfere with engraftment. Overall, the performance of TBF conditioning and PTCy use for HCT in MF patients has remained controversial so far, particularly in regard to engraftment dynamics. While no negative effect of TBF conditioning on engraftment has been shown in two recent studies (19, 50), the use of PTCy has been associated with significantly lower engraftment rates in one (19), but not in another study (17). Therefore, we believe it is important to amend these data from a major Swiss transplant center. Clearly, additional and larger studies are required to consolidate the findings of multivariate analyses in single center cohorts so far and to clarify, whether determinants of engraftment time in MF also affect graft function and overall outcome of MF patients in the longer term.

## Data availability statement

The data analyzed in this study is subject to the following licenses/restrictions: Retrospective analysis of electronic patient files. Requests to access these datasets should be directed to sara.meyer@insel.ch.

## Ethics statement

The studies involving humans were approved by Ethical committee Nordwest- und Zentralschweiz, Switzerland (EKNZ, no.2016-01930). The studies were conducted in accordance with the local legislation, institutional requirements and with the 1964 Helsinki Declaration and its later amendments or comparable ethical standards. The participants provided their written informed consent to participate in this study.

## Author contributions

SJ, JH and SM contributed to the study concept and design. SJ, KG, FA, MM, AB, and JP collected data, provided essential administrative or technical support, or analyzed and interpreted data. SJ and SM drafted the manuscript with contributions of all co-authors. All authors contributed to the article and approved the submitted version.
