# Supplementary material for: Characterization of engraftment dynamics in myelofibrosis after allogeneic hematopoietic cell transplantation including novel conditioning schemes
Source: Front Oncol. 2023 Aug 10;13:1205387. doi: 10.3389/fonc.2023.1205387 (PMC10449533; doi:10.3389/fonc.2023.1205387)
Supplement: Supplementary file 1 [file DataSheet_1.pdf]

Supplemental information to "Characterization of engraftment dynamics in myelofibrosis after allogeneic hematopoietic cell transplantation including novel conditioning schemes"

Sarah Jungius[1,2], Franziska C. Adam[3], Kerstin Grosheintz[3], Michael Medinger[3], Andreas Buser[3], Jakob R. Passweg[3], Jörg P. Halter[3*], Sara C. Meyer[1,2,3,4*]

[1]Department of Biomedicine, University Hospital Basel and University of Basel, Basel, Switzerland; [2] Department of Biomedical Research, University of Bern, Bern, Switzerland; [3]Division of Hematology, University Hospital Basel, Basel, Switzerland; [4]Department of Hematology and Central Hematology Laboratory, Inselspital, Bern University Hospital, University of Bern, Bern, Switzerland. *Co-corresponding authors.

Correspondence

Prof. Dr. med. Sara C. Meyer, Ph.D.

Email: sara.meyer@insel.ch

Supplemental Table 1

Patient and transplantation characteristics	First transplantation (n=60)	Second transplantation (n=11)
	n (%) unless otherwise specified	
**Time period**		
2000-2009	5 (8.3)	1 (9.1)
2010-2014	20 (33.3)	-
2015-2019	35 (58.3)	10 (90.9)
**Sex**		
male	36 (60)	7 (63.6)
female	24 (40)	4 (36.4)
**Age at transplantation**		
<55 years	19 (31.7)	1 (9.1)
55 - 64 years	27 (45.0)	5 (45.5)
>64 years	14 (23.3)	5 (45.5)
median age (range)	59.5 (31 - 70) years	60.0 (45 - 72) years
**Diagnosis**		
PMF	35 (58.3)	9 (81.8)
Secondary MF	23 (38.3)	2 (18.2)
PET-MF	*9 (15.0)*	-
PPV-MF	*14 (23.3)*	*2 (18.2)*
MPN/MDS overlap syndrome	1 (1.7)	-
MPN-U	1 (1.7)	-
**Driver mutation**		
JAK2 V617F	41 (68.3)	6 (54.5)
CALR	11 (18.3)	3 (27.3)
MPL	2 (3.3)	-
triple negative	2 (3.3)	1 (9.1)
n.a.	4 (6.7)	1 (9.1)
**Fibrosis grade**		
grade 1	1 (1.7)	1 (9.1)
grade 2	10 (16.7)	5 (45.5)
grade 3	36 (60)	4 (36.4)
n.a.	13 (21.7)	1 (9.1)
**Spleen**		
splenomegaly (≥13 cm)	55 (91.7)	10 (90.9)
no splenomegaly	3 (5.0)	-
prior splenectomy	2 (3.3)	1 (9.1)
median size (range)	19 (11 - 32) cm	16.75 (13 - 27) cm
**Ruxolitinib before HCT**		
no	25 (41.7)	8 (72.7)
yes	35 (58.3)	3 (27.3)
median time (range)	10 (1- 78.0) months	11.0 (1.0 - 12.0) months
**Donor relation**		
matched unrelated	31 (51.7)	4 (36.4)
matched related	21 (35.0)	5 (45.5)
haplo-identical	*6 (10.0)*	*1 (9.1)*
mismatched unrelated	2 (3.3)	-
n.a.	-	1 (9.1)

**Stem cell source**		
PBSC	53 (88.3)	9 (81.8)
BM	7 (11.7)	1 (9.1)
n.a.	-	1 (9.1)
**Stem cell dose**		
<6 x10^6 / kg	25 (41.7)	5 (45.5)
6 - 8 x10^6 / kg	13 (21.7)	2 (18.2)
>8 x10^6 / kg	21 (35.0)	3 (27.3)
n.a.	1 (1.7)	1 (9.1)
CD34+ cells (range)	7.2 (1.94 - 18.8) x10^6 / kg	6.575 (2.04 - 12.8) x10^6 / kg
**Conditioning regimen**		
RIC (incl. FluBu, FluTBI, FluMel)	32 (53.3)	8 (72.7)
MAC (incl. CyBu, CyTBI)	22 (36.7)	1 (9.1)
TBF	6 (10.0)	1 (9.1)
n.a.	-	1 (9.1)
**GvHD prophylaxis**		
CyA MTX +/- ATG	51 (85.0)	8 (72.7)
CyA MMF +/- PTCy	8 (13.3)	2 (18.2)
CyA	1 (1.7)	-
n.a.	-	1 (9.1)
**ATG**		
no	17 (28.3)	8 (72.7)
yes	43 (71.7)	2 (18.2)
n.a.	-	1 (9.1)
**PTCy**		
no	54 (90.0)	9 (81.8)
yes	6 (10.0)	1 (9.1)
n.a.	-	1 (9.1)
**G-CSF**		
no	50 (83.3)	7 (63.6)
yes	10 (16.7)	3 (27.3)
n.a.	-	1 (9.1)
**CMV risk**		
D- / R-	25 (41.7)	2 (18.2)
D+ / R+	17 (28.3)	3 (27.3)
D- / R+	10 (16.7)	4 (36.4)
D+ / R-	8 (13.3)	1 (9.1)
n.a.	-	1 (9.1)
**Blood-group barrier**		
no	41 (68.3)	7 (63.6)
minor	4 (6.7)	-
major	11 (18.3)	2 (18.2)
bidirectional	4 (6.7)	1 (9.1)
n.a.	-	1 (9.1)

Supplemental table 1: Baseline characteristics of patients' allogeneic hematopoietic cell transplantations for myelofibrosis. Characteristics of patients undergoing first (n=60) and second (n=11) allogeneic hematopoietic cell transplantations in 2000-2019 are indicated in absolute numbers, relative proportion in percent is indicated in parentheses. Continuous variables are indicated as median, the range is given in parentheses. n.a. not available, PMF primary myelofibrosis, MF myelofibrosis, PPV-/PET-MF post-polycythemia vera / post- essential thrombocythemia myelofibrosis, MPN-U unclassifiable myeloproliferative neoplasm, PBSC peripheral blood stem cells, BM bone marrow, RIC reduced-intensity conditioning, MAC myeloablative conditioning, TBF thiotepa-busulfan-fludarabin conditioning, GvHD graft versus host disease, CyA cyclosphorine A, MTX methotrexate, MMF mycophenolate mofetil, ATG anti-thymocyte globulin, PTCy post-transplantation cyclophosphamide, G-CSF granulocyte-colony stimulating factor, CMV cytomegalovirus, D/R donor / recipient.

Supplemental Table 2

Influencing factor		(%)	n	Neutrophil engraftment (d)	p-value	Platelet engraftment (d)	p-value	Reticulocyte engraftment (d)	p-value
**Splenomegaly** (HR, 95% CI)	Spleen (cm)	100	71	0.95 (0.906-0.996)	*0.032*	0.93 (0.89 - 0.98)	*0.002*	0.94 (0.90 - 0.99)	*0.008*
**Splenectomy** (Median, 95% CI)	no	95.8	68	20 (18.9 – 21.1)		27 (24.3 - 29.7)		21 (19.0 - 23.0)	
	yes	4.2	3	14 (n.a.)	*<0.001*	0 (n.a.)	*<0.001*	13 (11.4 - 14.6)	*0.005*
**Fibrosis grade** (Median, 95% CI)	grade 1	2.8	2	15 (n.a.)		19 (n.a.)		16 (n.a.)	
	grade 2	21.1	15	19 (18.1 – 19.9)	0.054	26 (22.6 - 29.4)	0.561	21 (19.2 - 22.8)	0.708
	grade 3	56.3	40	20 (18.9 – 21.1)	*0.017*	30 (28.0 - 32.0)	0.088	22 (18.9 - 25.1)	0.276
**CD34+ cell dose** (Median, 95% CI)	<6x10^6/kg	42.3	30	20 (18.7 – 21.3)		28 (24.4 - 31.6)		20 (16.8 - 23.2)	
	6-8x10^6/kg	21.1	15	21 (20.1 – 21.9)	0.895	30 (18.6 - 41.4)	0.395	25 (17.4 - 32.6)	0.362
	>8x10^6/kg	33.8	24	18 (16.4 – 19.6)	*0.037*	24 (21.6 - 26.4)	*0.021*	21 (16.2 - 25.8)	0.994
**CD34+ cell source** (Median, 95% CI)	PBSC	87.3	62	19 (17.8 – 20.2)		26 (23.2 - 28.8)		21 (18.7 - 23.3)	
	BM	11.3	8	26 (13.5 - 38.5)	*0.007*	30 (21.7 - 38.3)	0.148	24 (14.3 - 33.7)	0.212
**Conditioning** (Median, 95% CI)	TBF	9.9	7	21 (18.4 - 23.6)		48 (6.9 - 89.1)		28 (25.4 - 30.6)	
	RIC	56.3	40	20 (18.8 - 21.2)	*0.037*	25 (21.9 - 28.1)	*0.029*	20 (15.4 - 24.7)	0.243
	MAC	32.4	23	19 (15.5 - 22.5)	*0.033*	28 (25.2 - 30.8)	*0.018*	20 (18.0 - 22.0)	0.054
**Donor type** (Median, 95% CI)	haploidentical	9.9	7	21 (18.4 - 23.6)		48 (6.9 - 89.1)		28 (25.4 - 30.6)	
	HLA matched	85.9	61	20 (18.8 - 21.3)	*0.019*	26 (23.2 - 28.8)	*0.017*	20 (17.7 - 22.3)	0.118
	MR	36.6	26	21 (18.0 - 24.0)		24 (20.3 - 27.8)		20 (17.2 - 22.9)	
	MU	49.3	35	19 (17.9 - 20.2)	0.176	28 (24.4 - 31.6)	0.066	20 (17.1 - 22.9)	0.467
**ATG** (Median, 95% CI)	no	35.2	25	20 (18.4 - 21.6)		25 (18.5 - 31.5)		21 (16.1 - 25.9)	
	yes	63.4	45	19 (17.4 - 20.6)	*0.028*	28 (25.4 - 30.6)	0.853	21 (18.8 - 23.2)	0.758
**PTCy** (Median, 95% CI)	no	88.7	63	19 (17.8 - 20.2)		26 (24.3 - 27.7)		20 (17.9 - 22.1)	
	yes	9.9	7	21 (18.4 - 23.6)	*0.017*	48 (6.9 - 89.1)	*0.015*	28 (25.4 - 30.6)	0.126
**G-CSF** (Median, 95% CI)	no	80.3	57	19 (17.7 - 20.3)		26 (24.4 - 27.6)		20 (18.0 - 22.0)	
	yes	18.3	13	21 (17.5 - 24.5)	*0.004*	48 (18.1 - 77.9)	*0.001*	27 (20.0 - 34.1)	0.181

Supplemental table 2. Potential determinants of engraftment dynamics after allogeneic hematopoietic cell transplantation for myelofibrosis. Disease characteristics of myelofibrosis and transplant-related factors were assessed for potential influence on reconstitution times of neutrophil, platelet and reticulocyte lineages. Effects of splenomegaly were analyzed using spleen size (cm) as a continuous variable, since splenomegaly was present in all but one patient, and effects on engraftment (days post-transplant) indicated as hazard ratio (HR) incl. 95% confidence interval (CI) in brackets. For all other factors, median time to engraftment is indicated in days post-transplant (d) with 95% confidence interval (CI) in brackets. P-values were determined by Log-Rank test with values <0.05 considered as statistically significant (italics). n absolute number of transplantations, n.a. not available, PBSC peripheral blood stem cells, BM bone marrow, TBF thiotepa-busulfan-fludarabin, RIC reduced-intensity conditioning, MAC myeloablative conditioning, MR matched related, MU matched unrelated, ATG antithymocyte globulin, PTCy post-transplantation cyclophosphamide, G-CSF granulocyte-colony stimulating factor.

Supplemental Table 3

Time to neutrophil engraftment		HR	95% CI	p-value
G-CSF		**0.415**	**0.198 - 0.870**	**0.02**
PBSC vs BM		**0.421**	**0.185 - 0.959**	**0.039**
Time to platelet engraftment		HR	95% CI	p-value
Spleen size		**0.94**	**0.892 - 0.991**	**0.021**
G-CSF		**0.301**	**0.140 - 0.646**	**0.002**
Fibrosis grade	1 (reference)			**0.021**
	2	0.421	0.091 - 1.941	0.267
	3	**0.179**	**0.039 - 0.815**	**0.026**
	missing	0.221	0.045 - 1.083	0.063
Time to reticulocyte engraftment		HR	95% CI	p-value
Spleen size		**0.942**	**0.900 - 0.987**	**0.012**
Blood group barrier	no (reference)			**0.028**
	minor	2.076	0.731 - 5.891	0.17
	major	**0.449**	**0.230 - 0.878**	**0.019**
	bidirectional	1.658	0.580 - 4.739	0.345

Supplemental table 3. Multivariate analyses for determinants of time to neutrophil, platelet, and reticulocyte engraftment. All variables significant in univariate analyses except splenectomy, which was excluded due to very low patient number with this procedure, were included in the analyses. Significant values are highlighted in bold.

Supplemental Table 4

**Influencing factor**		**Neutrophil engraftment**		**Platelet engraftment**		**Reticulocyte engraftment**	
		Univariate	Multivariate	Univariate	Multivariate	Univariate	Multivariate
Splenomegaly	Spleen (cm)	0.032	n.s.	0.002	0.021	0.008	0.012
Splenectomy	yes / no	<0.001	n.a.	<0.001	n.a.	0.005	n.a.
Fibrosis grade	grade 1				0.021		
	grade 2	0.054	n.s.	0.561	0.267	0.708	
	grade 3	0.017		0.088	0.026	0.276	n.s.
CD34+ cell dose	<6x10^6/kg						
	6-8x10^6/kg	0.895	n.s.	0.395	n.s.	0.362	n.s.
	>8x10^6/kg	0.037		0.021		0.994	
CD34+ cell source	BM / PBSC	0.007	0.039	0.148	n.s.	0.212	n.s.
Conditioning	TBF				n.s.		
	RIC	0.037		0.029		0.243	
	MAC	0.033	n.s.	0.018		0.054	n.s.
Donor type	haplo / HLA matched	0.019	n.s.	0.017	n.s.	0.118	n.s.
	MR / MU	0.176	n.s.	0.066	n.s.	0.467	n.s.
ATG	yes / no	0.028	n.s.	0.853	n.s.	0.758	n.s.
PTCy	yes / no	0.017	n.s.	0.015	n.s.	0.126	n.s.
G-CSF	yes / no	0.004	0.020	0.001	0.002	0.181	n.s.
Blood group barrier	no						0.028
	minor	0.547		0.504		0.107	0.170
	major	0.234	n.s.	0.883	n.s.	0.032	0.019
	bidirectional	0.276		0.290		0.454	0.345

Supplemental table 4. Univariate and multivariate analyses of factors influencing engraftment dynamics. P values of univariate and multivariate analyses of MF disease characteristics and transplant-related factors with potential influence on engraftment dynamics are indicated. Effects of splenomegaly were analyzed using spleen size (cm) as a continuous variable with Cox regression analysis, since splenomegaly was present in all but one patient. Splenectomy was excluded as a factor from multivariate analysis since only two patients of the entire cohort underwent allogeneic HCT with absent spleen. Unless otherwise specified, univariate analysis was performed with Kaplan-Meier method and analyzed with log-rank test. Multivariate analysis was performed with forward-conditional Cox regression. P-values <0.05 were considered statistically significant. n.s. not significant, n.a. not available, BM bone marrow, PBSC peripheral blood stem cells, TBF thiotepa-busulfan-fludarabin, RIC reduced-intensity conditioning, MAC myeloablative conditioning, MR matched related, MU matched unrelated, ATG antithymocyte globulin, PTCy post-transplantation cyclophosphamide, G-CSF granulocyte-colony stimulating factor.

Supplemental Table 5

Pat No.	HCT period	Age (y)	Sex	Diagnosis	Driver	HCT	Onset (d)	Neu	Plt	Ret	Duration (d)	Spleen (cm)	Fibrosis	Donor	Conditioning	Cells (Mio/kg)	Source	BG barrier
1	2010-2014	67	f	PMF	CALR	1st	58	x	x	x	63	17	grade 3	MU	RIC	8.85	PBSC	major
2	2010-2014	46	f	PET-MF	JAK2 V617F	1st	39	x	x	x	40	15	grade 3	MR	MAC	3.79	PBSC	major
3	2010-2014	65	m	PMF	CALR	1st	39	x	x	x	49	16	n.a.	MU	RIC	7.07	PBSC	major
4	2010-2014	70	m	PMF	CALR	1st	29	x	x	x	52	21	n.a.	MU	RIC	5.2	PBSC	none
5	2015-2019	63	f	PMF	JAK2 V617F	1st	95	x	x	x	203	21.5	grade 3	MU	RIC	7.86	PBSC	major
6	2015-2019	57	f	PMF	CALR	1st	36	x	x	x	187	18	grade 3	Haplo	TBF	3.772	BM	none
7	2015-2019	68	m	PMF	JAK2 V617F	1st	73	x	-	x	21	27	grade 1-2	MU	RIC	4.94	PBSC	major
8	2015-2019	60	f	PMF	CALR	2nd	29	x	x	x	90	17.5	grade 3	MU	RIC	7.5	PBSC	none

Supplemental table 5. Characteristics of patients with poor graft function. Poor graft function as defined previously by McLornan 2021, Lavi 2017 and Kröger 2016 was assessed in MF patients within 6 months after allogeneic HCT. Characteristics of the n=8 patients with poor graft function are indicated. PMF: primary myelofibrosis, PET-MF: post-essential thrombocythemia myelofibrosis, Neu: neutrophil granulocytes, Plt: platelets, Ret: reticulocytes, MU: matched unrelated, MR: matched related, Haplo: haploidentical, RIC: reduced-intensity conditioning, MAC: myeloablative conditioning, TBF: thiotepa-busulfan-fludarabin conditioning, PBSC: peripheral blood stem cell, BM: bone marrow, BG: blood group.

Supplemental Figure 1

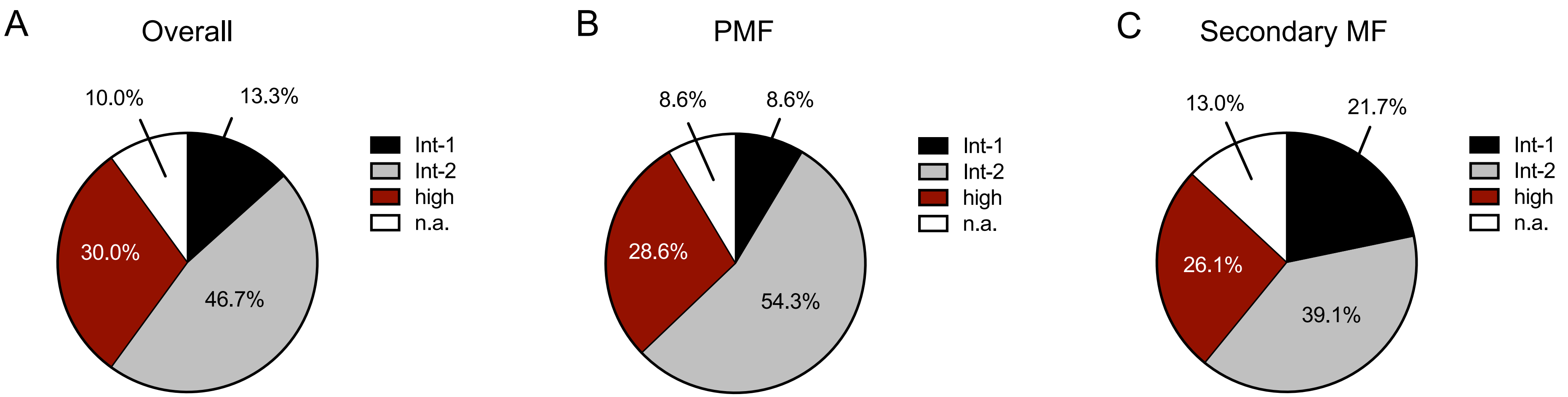

Supplemental figure 1. Characterization of MF patients' prognostic risk. The prognostic risk group is indicated for the overall MF patient cohort (**A**) as well as specifically for patients with PMF (**B**) or secondary MF (**C**) at first allogeneic HCT. Since patients were included between 2000-2019, several risk scores including DIPSS plus, DIPSS, IPSS Cervantes score, MIPSS70 plus or MYSEC sore were used to determine low, intermediate-1, intermediate-2 or high risk groups. Second HCT were not considered given their potentially different situation regarding disease risk.

Supplemental Figure 2

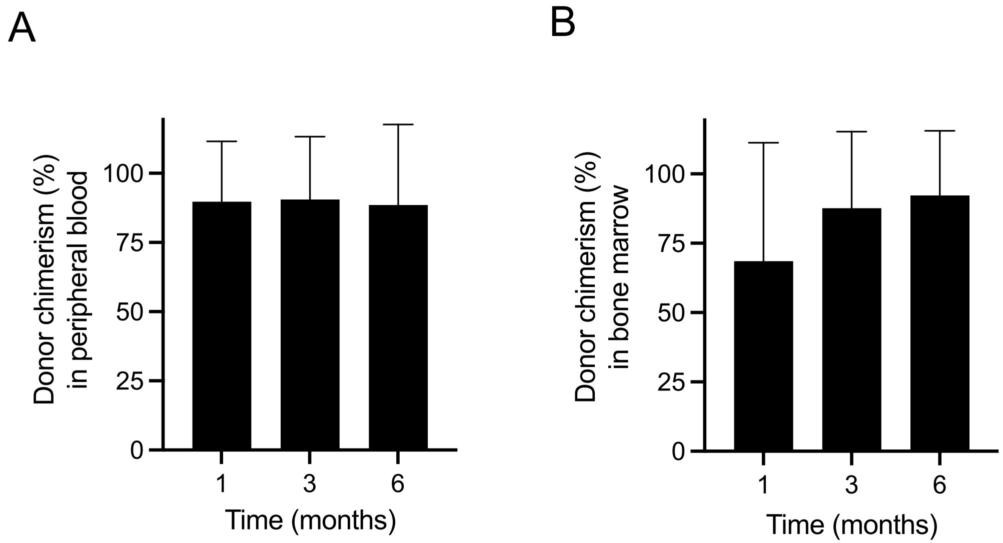

Supplemental figure 2. Donor cell chimerism after allogeneic HCT for myelofibrosis. A. Donor cell chimerism in peripheral blood was determined at 1, 3 and 6 months after HCT with median chimerism of 100% and mean of 90%, 91% and 89%, respectively, without significant differences. Mean +/- SD is shown in the graph. **B.** Donor cell chimerism in bone marrow was determined at 1,3 and 6 months after HCT, while analysis at the 1-month time-point contains limited data (n=8 patients). Mean +/- SD is shown in the graph. HCT hematopoietic cell transplantation.

Supplemental Figure 3

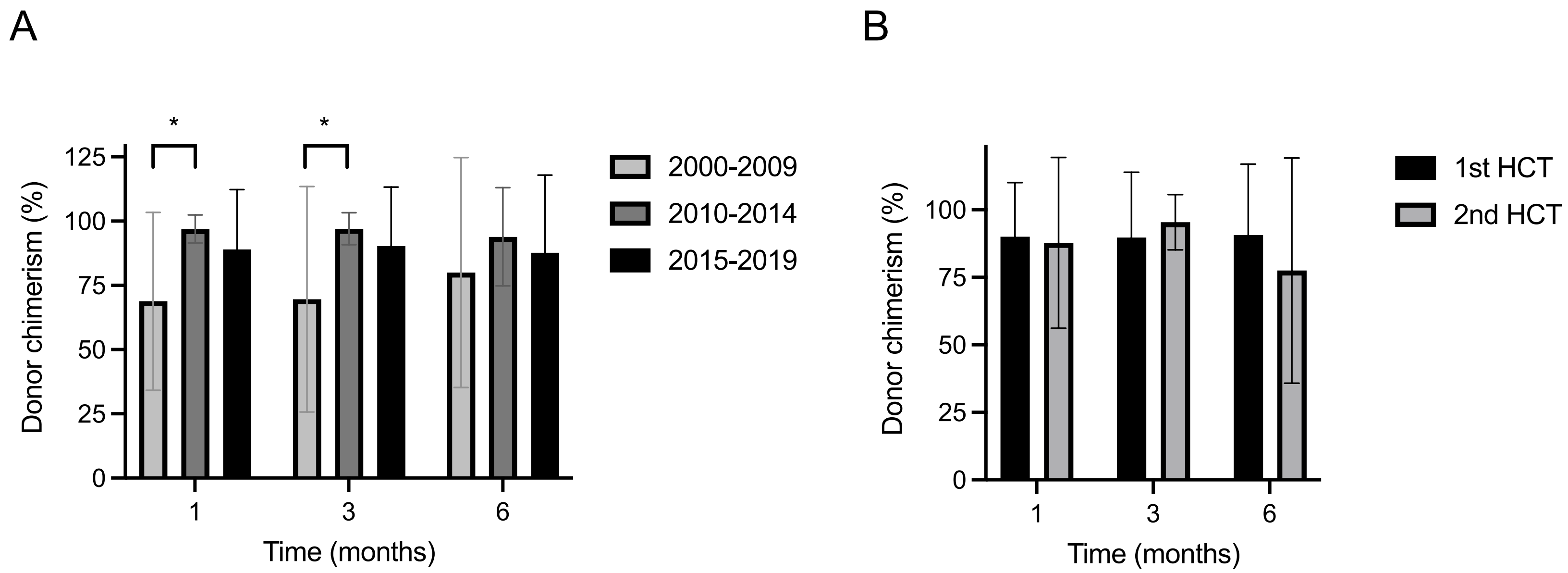

Supplemental figure 3. Characteristics of donor cell chimerism. A. Donor cell chimerism at 1, 3 and 6 months after HCT was assessed in the three time periods 2000-2009, 2000-2014 and 2015-2019. **B.** Donor cell chimerism was characterized at 1, 3 and 6 months after HCT without significant differences after first and second HCT. One-way ANOVA with Tukey correction or Mann-Whitney U-test were used for statistical analysis. * $p < 0.05$ considered statistically significant. HCT hematopoietic cell transplantation.

Supplemental Figure 4

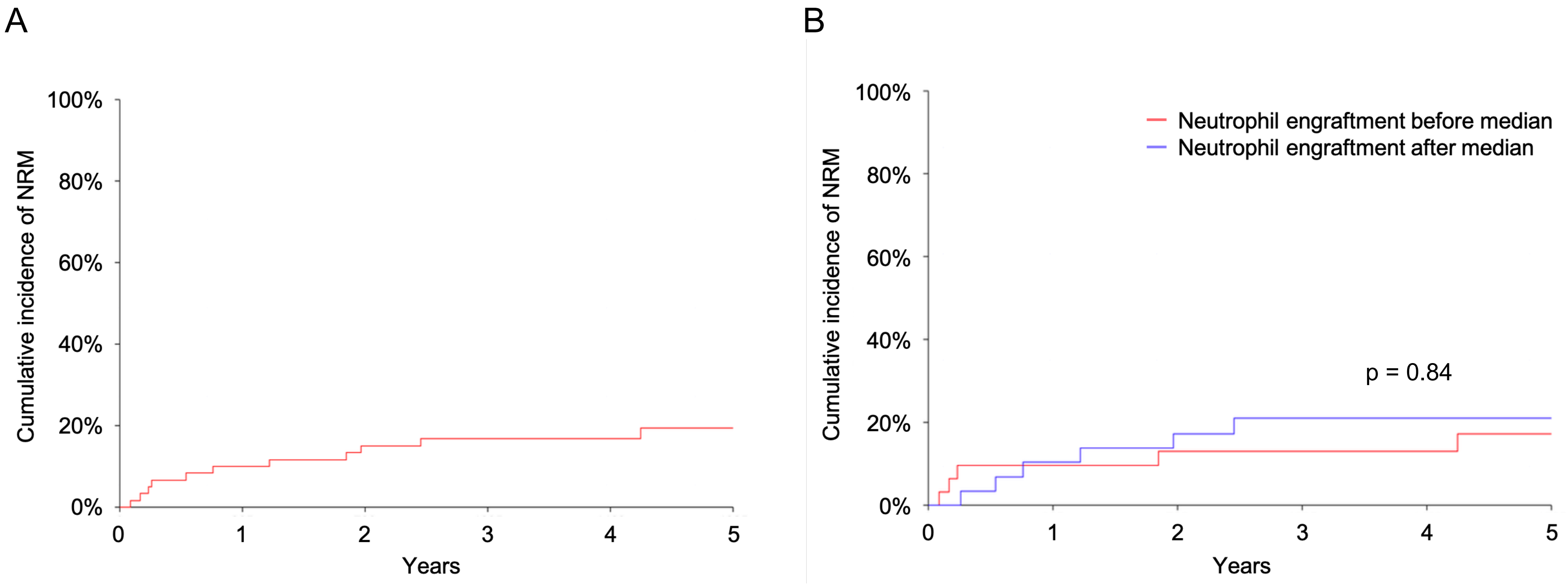

Supplemental figure 4. Non-relapse mortality (NRM) after allogeneic HCT for myelofibrosis. A. Cumulative incidence of NRM at 5 years after allogeneic HCT is indicated as assessed by competitive risk analysis with relapse as competitive event. **B.** Cumulative incidence of NRM is assessed for patients with neutrophil engraftment time before vs. after the median engraftment time. In our cohort, delayed engraftment did not significantly affect cumulative incidence of NRM. * $p < 0.05$ considered statistically significant. HCT hematopoietic cell transplantation.

Supplemental Figure 5

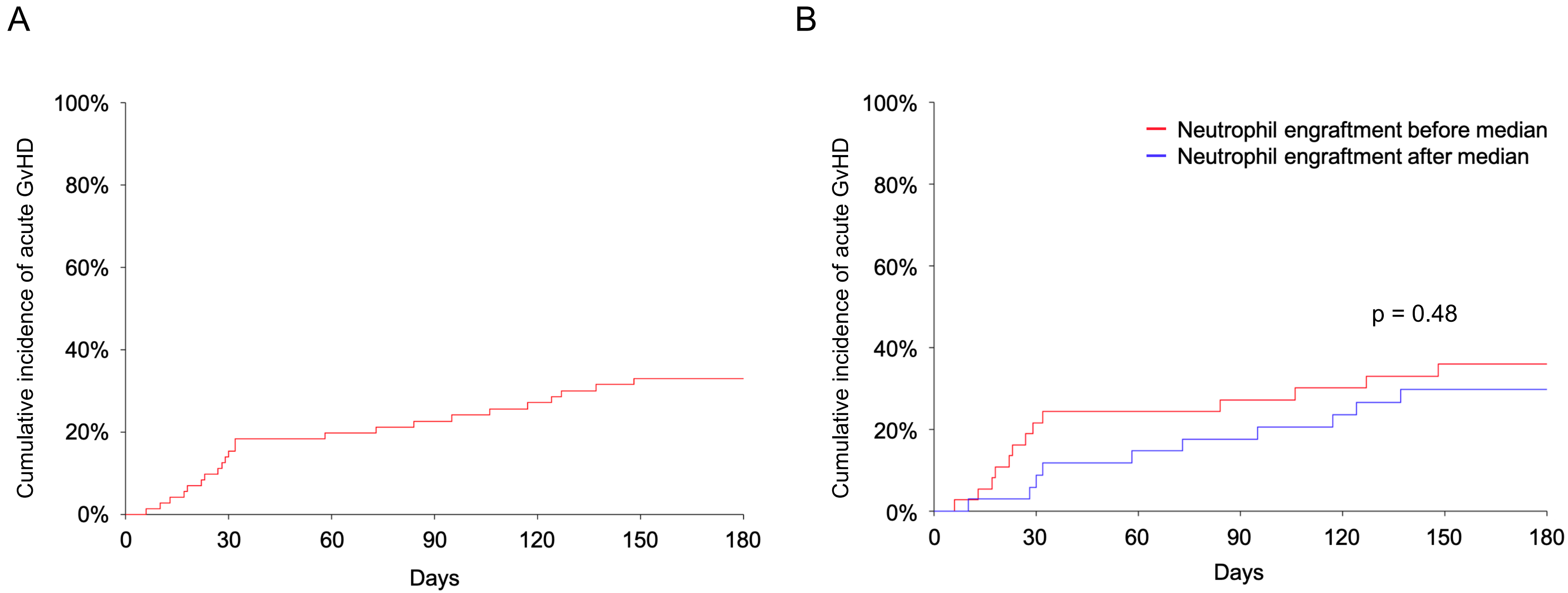

Supplemental figure 5. Acute Graft versus Host Disease (aGvHD) after allogeneic HCT for myelofibrosis. A. Cumulative incidence of aGvHD with grades 2-4 at 180 days after HCT is indicated as assessed by competitive risk analysis with relapse as competing event. **B.** Cumulative incidence of aGvHD is assessed for patients with neutrophil engraftment time shorter vs. longer than the median engraftment time. In our cohort, delayed engraftment did not significantly affect cumulative incidence of aGvHD. * $p < 0.05$ considered statistically significant. HCT hematopoietic cell transplantation.

Supplemental Figure 6

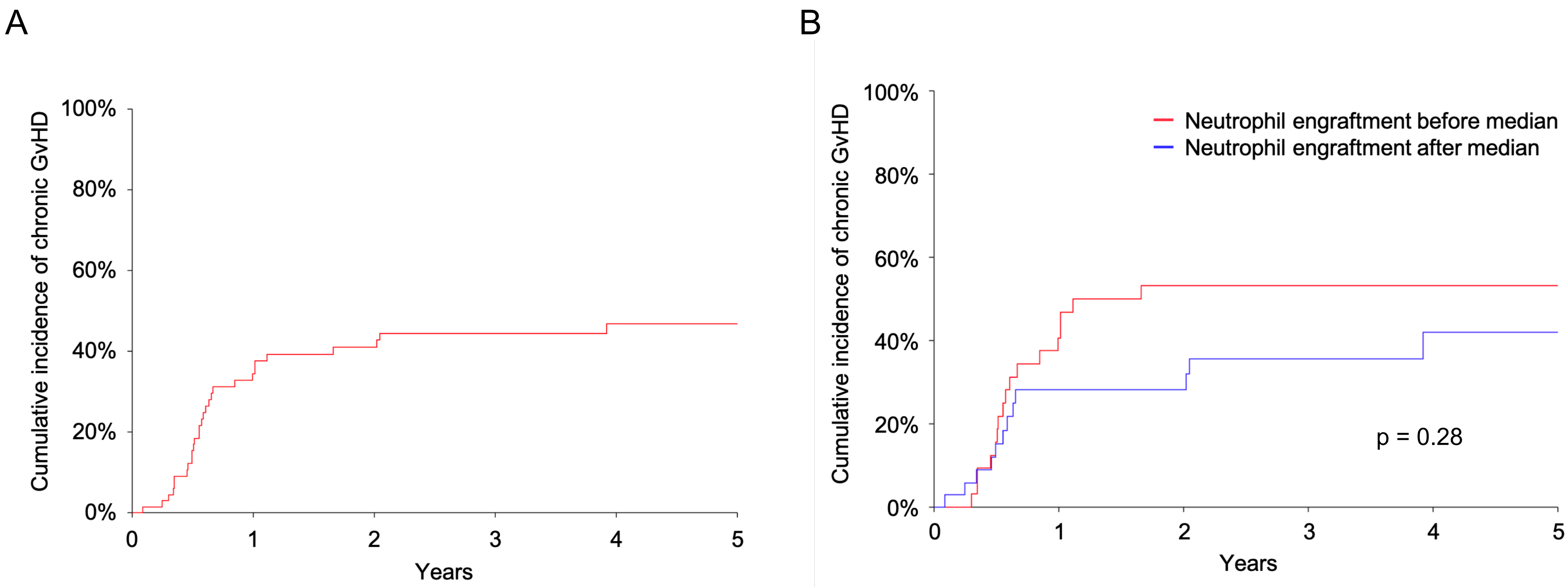

Supplemental figure 6. Chronic Graft versus Host Disease (cGvHD) after allogeneic HCT for myelofibrosis. A. Cumulative incidence of cGvHD as assessed at 5 years after HCT is indicated as assessed by competitive risk analysis with relapse as competing event. **B.** Cumulative incidence of cGvHD is assessed for patients with neutrophil engraftment time shorter vs. longer than the median engraftment time. In our cohort, delayed engraftment did not significantly affect cumulative incidence of cGvHD. * $p < 0.05$ considered statistically significant. HCT hematopoietic cell transplantation.

Supplemental Figure 7

A

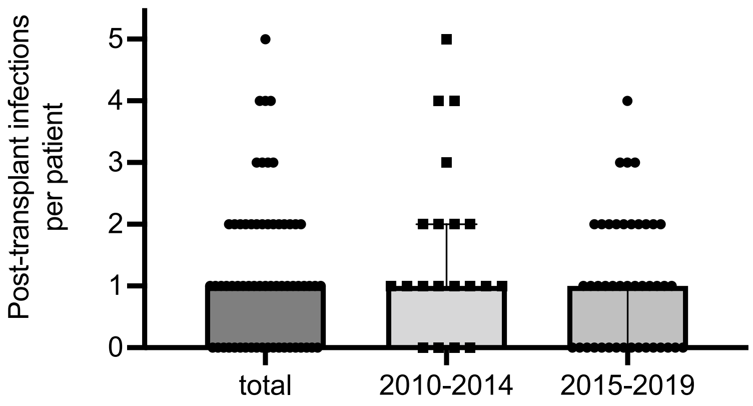

B

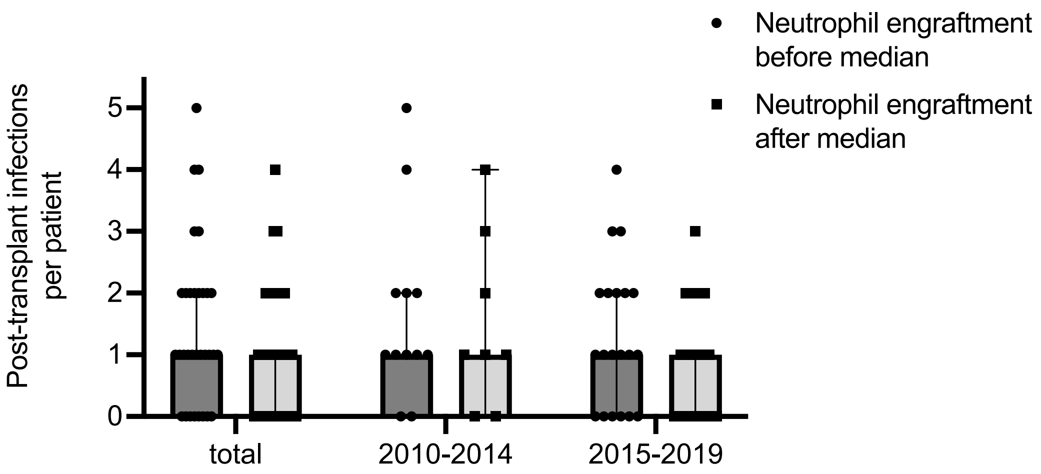

Supplemental figure 7. Post-transplant infections after allogeneic HCT for myelofibrosis. The number of infections per patient with need for hospitalization within 6 months after allogeneic HCT was assessed from 2010 onwards. **A.** A median of one infection (range 0-5 infections) occurred per patient within 6 months both in the 2010-2014 and 2015-2019 time periods. **B.** Delayed engraftment when assessed as engraftment time longer than the median of the cohort, associated with similar numbers of infections per patient within 6 months of HCT.

Supplemental Figure 8

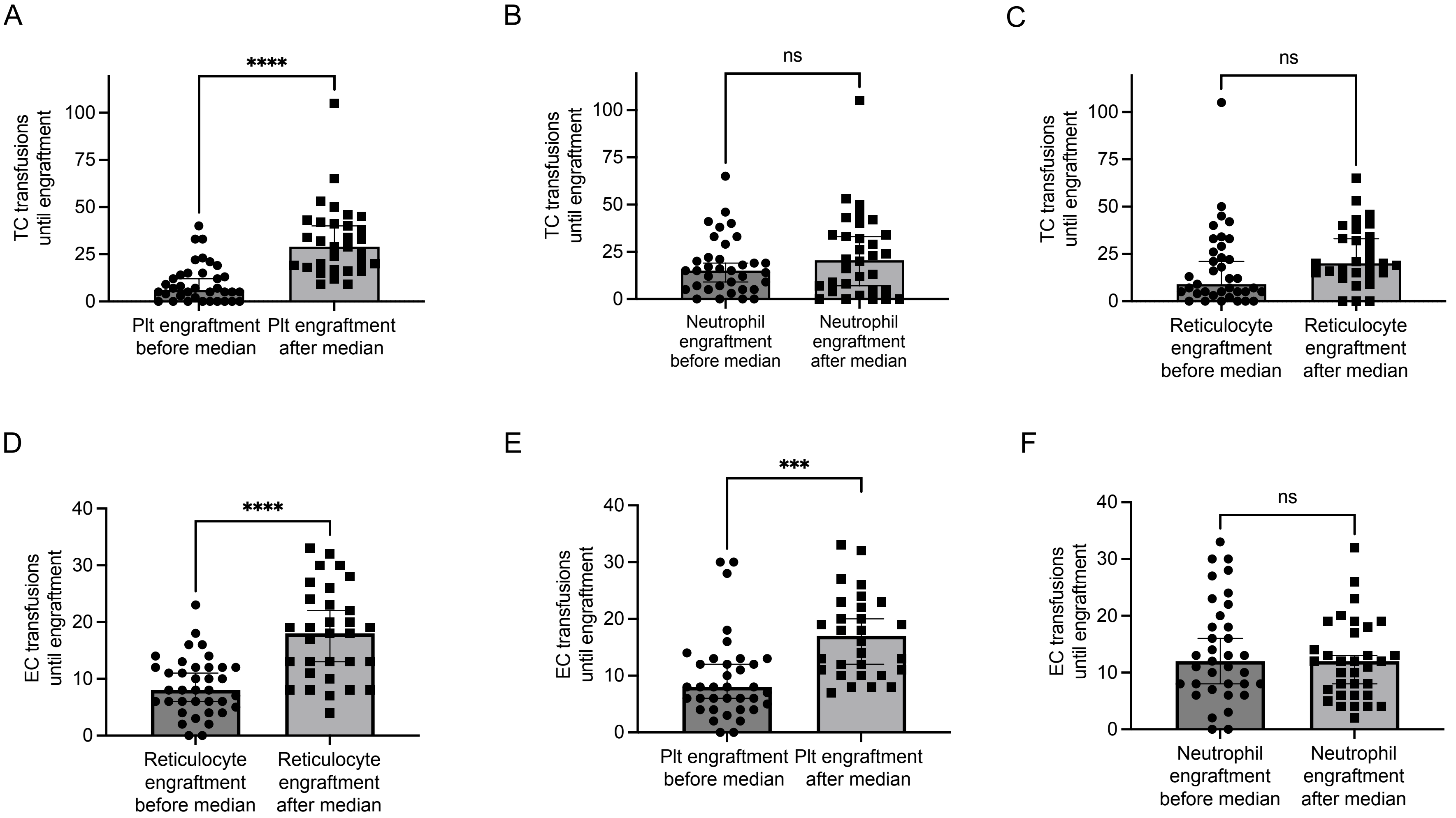

Supplemental figure 8. Thrombocyte and erythrocyte transfusion requirements until engraftment. The number of thrombocyte concentrate (TC) transfusions and erythrocyte concentrate (EC) transfusions until engraftment are shown from 2000-2019. Delayed platelet engraftment when assessed as engraftment time longer than the median of the cohort, associated with increased TC transfusion requirement (**A**), while neutrophil (**B**) and reticulocyte (**C**) engraftment dynamics did not show an impact. Delayed reticulocyte as well as platelet engraftment when assessed as engraftment time longer than the median of the cohort, associated with increased EC transfusion requirement (**D-E**), while neutrophil engraftment dynamics did not show an impact (**F**).
* p <0.05 considered statistically significant, *** p<0.001, **** p <0.0001.

Supplemental Figure 9

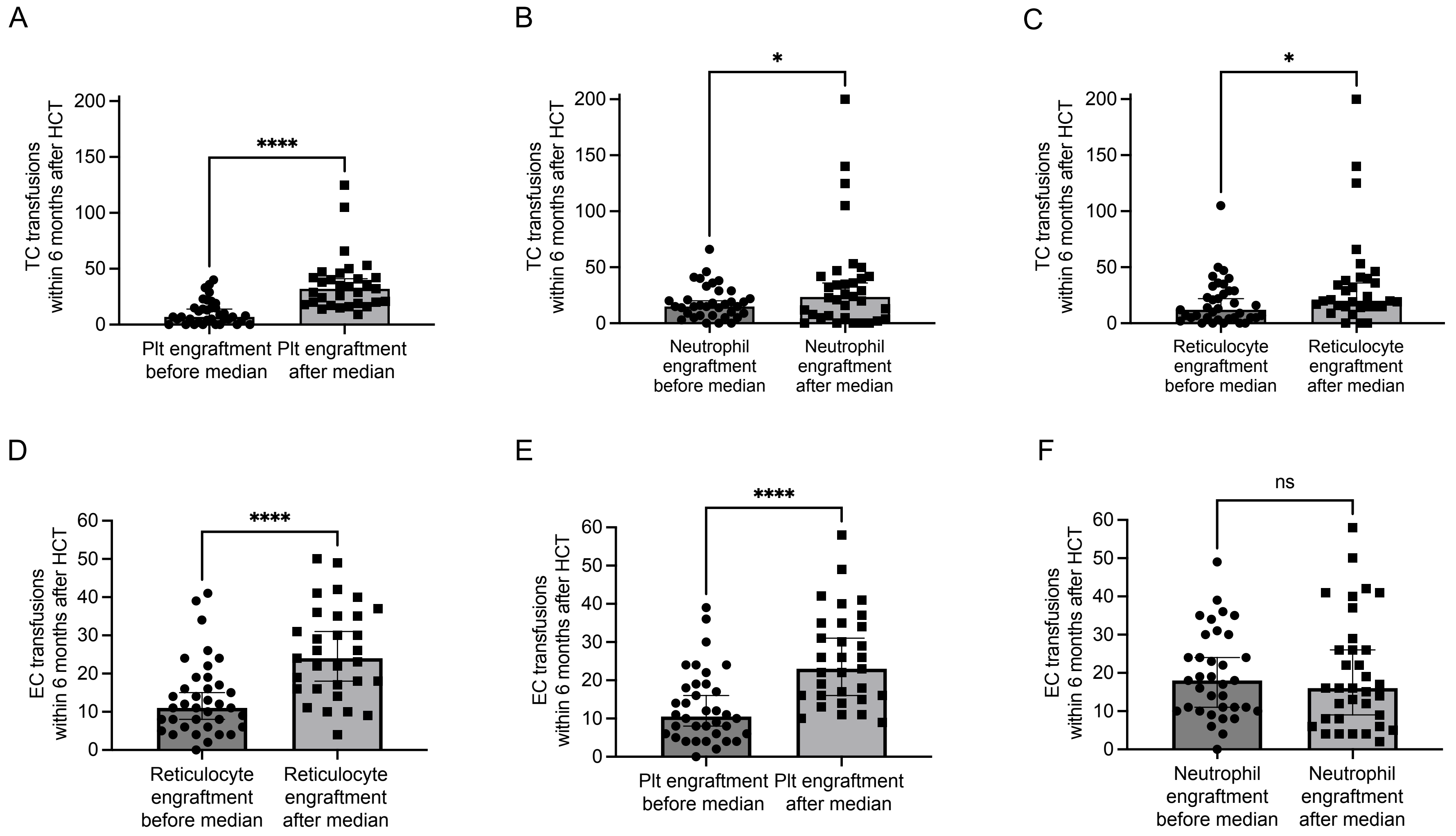

Supplemental figure 9. Thrombocyte and erythrocyte transfusion requirements within 6 months after HCT. The number of thrombocyte concentrate (TC) transfusions and erythrocyte concentrate (EC) transfusions within 6 months after HCT are shown from 2000-2019. Delayed platelet and neutrophil engraftment when assessed as engraftment time longer than the median of the cohort, associated with increased TC transfusion requirement (**A**), while also neutrophil (**B**) and reticulocyte (**C**) engraftment dynamics had a certain, although more modest, impact. Delayed reticulocyte as well as platelet engraftment when assessed as engraftment time longer than the median of the cohort, associated with increased EC transfusion requirement (**D-E**), while neutrophil engraftment dynamics did not show an impact (**F**). * $p < 0.05$ considered statistically significant, **** $p < 0.0001$. HCT hematopoietic cell transplantation.
